# Sus1, Cdc31, and the Sac3 CID Region Form a Conserved Interaction Platform that Promotes Nuclear Pore Association and mRNA Export

**DOI:** 10.1016/j.molcel.2009.01.033

**Published:** 2009-03-27

**Authors:** Divyang Jani, Sheila Lutz, Neil J. Marshall, Tamás Fischer, Alwin Köhler, Andrew M. Ellisdon, Ed Hurt, Murray Stewart

**Affiliations:** 1 MRC Laboratory of Molecular Biology, Hills Road, Cambridge CB2 0QH, UK; 2 Biochemie-Zentrum der Universität Heidelberg, INF328, D-69120 Heidelberg, Germany

**Keywords:** PROTEINS, RNA

## Abstract

The yeast Sac3:Cdc31:Sus1:Thp1 (TREX-2) complex facilitates the repositioning and association of actively transcribing genes with nuclear pores (NPCs)—“gene gating”—that is central to integrating transcription, processing, and mRNA nuclear export. We present here the crystal structure of Sus1 and Cdc31 bound to a central region of Sac3 (the CID domain) that is crucial for its function. Sac3^CID^ forms a long, gently undulating α helix around which one Cdc31 and two Sus1 chains are wrapped. Sus1 has an articulated helical hairpin fold that facilitates its wrapping around Sac3. In vivo studies using engineered mutations that selectively disrupted binding of individual chains to Sac3 indicated that Sus1 and Cdc31 function synergistically to promote NPC association of TREX-2 and mRNA nuclear export. These data indicate Sac3^CID^ provides a scaffold within TREX-2 to integrate interactions between protein complexes to facilitate the coupling of transcription and mRNA export during gene expression.

## Introduction

There is an emerging consensus that transcription, pre-mRNA processing, and the export of mature mRNA to the cytoplasm are coupled (Strässer et al., 2002; Reed and Hurt, 2002; Stutz and Izaurralde, 2003; Rodriguez-Navarro et al., 2004; Masuda et al., 2005; Aguilera, 2005). This integration of the gene expression machinery relies on a complex network of interactions between activated genes, processing factors, and the nuclear pore complex (NPC). In many instances, members of the transcription and mRNA export machinery associate with NPC components to integrate the steps of gene expression (Casolari et al., 2004; Brown and Silver, 2007), a process referred to as “gene gating” (Blobel, 1985) and in which Sac3, Sus1, and Cdc31 have a central function (Rodriguez-Navarro et al., 2004; Fischer et al., 2004; Cabal et al., 2006).

The export of mRNA to the cytoplasm through NPCs is mediated by the Mex67:Mtr2 heterodimer in yeast and NXF1:NXT1 (TAP:P15) in metazoans (Stutz and Izaurralde, 2003; Reed and Hurt, 2002; Köhler and Hurt, 2007). Mex67:Mtr2 is recruited to mRNA during transcription and processing via proteins, including Yra1 and Sub2. Once associated with the mRNP, Mex67:Mtr2 interacts with the phenylalanine-glycine (FG) repeats present in many nuclear pore proteins (nucleoporins) to facilitate transport through NPCs. Studies in yeast and mammals show that Yra1 and Sub2 are part of the multisubunit TREX (*TR*anscription and *EX*port) complex, which also contains components (THO) involved in transcription elongation (Chávez et al., 2000; Strässer et al., 2002; Hurt et al., 2004).

A genetic screen with Yra1 identified an additional transcription and mRNA export complex, TREX-2, consisting of Sac3, Thp1, Sus1, and Cdc31 (Fischer et al., 2002, 2004). Sac3 is the central component of TREX-2 and contains a conserved Sac3/germinal center-associated nuclear protein (GANP) domain, which, along with the short N domain, binds both Mex67:Mtr2 and Thp1, a factor involved in transcription elongation. The Sac3 C domain is responsible for NPC association that is lost in strains in which nucleoporin Nup1 is deleted (Fischer et al., 2002). Electron microscopy has also detected Sac3 at the NPC cytoplasmic fibrils (Lei et al., 2003). A region within the Sac3 C region (Sac3^CID^) binds Sus1 and the calmodulin-like protein, Cdc31 (Fischer et al., 2004). Deletion of the Sac3 CID domain results in a loss of Sus1 and Cdc31 from TREX-2, coupled with a defect in poly(A)^+^ RNA export and a mislocalization of TREX-2 from NPCs (Fischer et al., 2004). Sac3 has also been proposed to be involved in nuclear protein export (Jones et al., 2000) and is required for normal progression of mitosis (Bauer and Kölling, 1996).

Sus1 is also a component of SAGA, a large complex involved in chromatin remodeling and transcription activation. Within SAGA, Sus1, together with Sgf11 and Sgf73, is required for activation of the histone H2B deubiquitinating protease, Ubp8 (Köhler et al., 2008). SAGA and TREX-2 have physical and functional links. Significantly, deletions of components of SAGA, TREX-2, or Nup1 cause defects in the movement of *GAL* genes upon transcriptional activation to the nuclear periphery, a process fundamental to gene gating (Cabal et al., 2006). Moreover, mutations in Sgf73 were found to affect the stability of TREX-2 (Köhler et al., 2008). Although the precise way in which SAGA and TREX-2 are linked physically is unknown, this interaction is mediated by Sgf73 and potentially Sus1 (Rodriguez-Navarro et al., 2004; Köhler et al., 2006).

We describe here structure-function relationships involving the Sac3-CID motif and its role in transcription-coupled mRNA export. We identify the Sus1 and Cdc31 binding sites on Sac3 and present the crystal structure of the Sac3^CID^:Sus1:Cdc31 complex. The CID region comprises Sac3 residues 723–805 and forms a gently undulating, continuous 12.5 nm α helix, which is encircled by two Sus1 chains and one Cdc31 chain. Sus1 has an articulated α-hairpin fold that is constructed from five α helices connected by highly conserved hinges that are crucial for wrapping around Sac3. Mutant phenotypes and genetic interactions in yeast of Sac3^CID^ indicate that Sus1, Cdc31, and Sac3 function synergistically to promote efficient mRNA export. Moreover, the CID motif is targeted to NPCs, and Sus1 is necessary for this targeting. Taken together, our data indicate Sac3^CID^ functions as a conserved α-helical scaffold that, together with Sus1 and Cdc31, is necessary for TREX-2 function.

## Results

### Sac3^CID^ Contains Two Sus1 Binding Sites in the TREX-2 Complex

**Figure 1:**
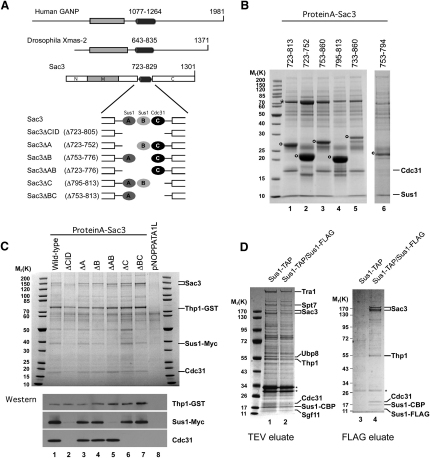
Dissection of the Interactions between Sac3, Sus1, and Cdc31 in the TREX-2 Complex (A) Schematic representation of Sac3 and its homologs, human GANP and *Drosophila* Xmas-2. The predicted α helix (dark gray cylinder) is located downstream of the conserved Sac3/GANP PFAM domain, PF03399 (gray rectangle). Sac3 mutants within the α-helical region are depicted below with the Sus1 and Cdc31 binding sites inferred from the purifications in (B), (C), and (D). (B) Cell lysates from *sac3Δ* cells containing overexpressed Protein A-tagged *SAC3* fragments (under *GAL1* promoter control) were affinity purified over IgG Sepharose. The acid-released eluates were analyzed by SDS-PAGE and Coomassie staining. Open circles indicate Sac3 fragments. The asterisk indicates, Ssa1, and Ssa2, likely contaminants, identified by mass spectrometry. (C) Split-tag affinity purification of the TREX-2 complex. Plasmid-based (pNOPPATAIL) Protein-A-tagged Sac3 or mutant derivatives were expressed in *sac3Δ*, *THP1*-GST, and *SUS1*-MYC cells and affinity purified over IgG Sepharose. After TEV cleavage, the eluate was further purified over GSH Sepharose and eluted with glutathione. The final eluate was analyzed by SDS-PAGE and Coomassie staining, (upper panel) or western blotting with anti-GST (to detect Thp1), anti-myc (to detect Sus1), or anti-Cdc31 antibodies (lower panel). (D) Protein A/Flag split purification of Sus1. Sus1-TAP (via the Protein A tag) was first affinity purified on IgG Sepharose from a yeast strain expressing Sus1-TAP alone or a diploid strain expressing Sus1-TAP and Sus1-FLAG. After TEV cleavage, the eluates (lanes 1 and 2) were subject to Sus1-FLAG purification by anti-FLAG M2 affinity resin and eluted with free FLAG peptide. All eluates were analyzed by SDS-PAGE and Coomassie staining. Indicated copurifying proteins were identified by mass spectrometry. Asterisks denote TEV protease.

Previous work identified a region of Sac3 (residues 733–860, the CID motif) that bound Sus1 and Cdc31 and which had a dominant-lethal phenotype when overexpressed (Fischer et al., 2004). Analysis of the CID region for conserved sequence and structural patterns indicated a high probability that Sac3 residues 723–829 formed an extended α helix, which also was predicted in homologous proteins, including human GANP and *Drosophila* Xmas2 (Figure 1A). Alignment of the conserved α-helical motifs (see Figure S1 available online) indicated short regions of higher conservation, including a putative Cdc31 binding site encompassing Sac3 residues 795–813 and a putative binding site for Sus1 involving residues ∼760–790.

Fragments of the CID motif and α-helical region of Sac3 encompassing residues 723–860 were overexpressed in yeast and affinity purified (Figure 1B). Sus1 and Cdc31 copurified with both the original CID motif and residues 723–813 (lanes 1 and 5). Further dissection showed that Cdc31 but not Sus1 copurified with Sac3 residues 795–813 (lane 4), whereas Sus1 but not Cdc31 copurified with residues 723–752 and 753–794 (lanes 2 and 6). These results indicated that Sus1 and Cdc31 can bind to Sac3 independently of one another and that Sac3 may contain two Sus1 binding sites. Furthermore, overexpression of all Sac3 fragments that contained the binding site for Cdc31, but not those for Sus1, was toxic to yeast, supporting the hypothesis that the CID overexpression toxicity was due to its sequestering Cdc31, preventing it from performing its essential role in mitosis (Figure S2; Fischer et al., 2004).

We complemented these experiments using engineered *sac3* mutants in which the regions encompassing each of the putative Sus1 and Cdc31 binding sites were deleted (Figure 1A). The TREX-2 complex was affinity purified using a split-tag protocol from yeast strains expressing protein A-tagged Sac3 or mutant derivatives and Thp1-GST. Copurification of Myc-tagged Sus1 and Cdc31 was detected by western blotting (Figure 1C). Sus1 and Cdc31 copurified with wild-type Sac3 (lane 1), whereas deletion of Sac3 residues 723–805 (Sac3ΔCID) abolished binding to both Sus1 and Cdc31 (lane 2), indicating that there were no additional binding sites for these proteins on Sac3. In vitro studies (Figure S3) indicated residues 806–813 were not necessary for Cdc31 binding. When either putative Sus1 binding site was deleted (Sac3ΔA or Sac3ΔB), Sus1 still copurified with the TREX-2 complex (Figure 1C, lanes 3 and 4), whereas deletion of both Sus1 binding sites (Sac3ΔAB) abolished Sus1 association but retained Cdc31 binding (lane 5). This demonstrated that Sus1 could probably bind independently to each site in the TREX-2 complex. Additionally, deletion of the putative Cdc31 binding site (Sac3ΔC) abolished copurification of Cdc31 with Sac3 but retained Sus1 binding (lane 6). Because the deletion mutants retained affinity for the other binding partners, it is unlikely that the loss of copurification was caused by an alteration of the overall structure or stability of Sac3.

### Sus1 Is Present in More Than One Copy in TREX-2 but Not in SAGA

The copurification of Sus1 with two nonoverlapping regions of Sac3 indicated that either Sac3 binds two Sus1 molecules or that one Sus1 binds to two adjacent sites on Sac3. To determine if more than one Sus1 chain is present in the TREX-2 complex, Sus1 was split-tag affinity purified from a diploid yeast strain in which one copy of Sus1 carried a TAP tag and the other carried a FLAG tag (Figure 1D). When Sus1-TAP was isolated via the protein A tag, both SAGA and TREX-2 (lanes 1 and 2) copurified, but when this material was subsequently purified via Sus1-FLAG, only TREX-2 copurified (lane 4). Crucially, the selective recovery of TREX-2 in the FLAG affinity purification step was not due to nonspecific TREX-2 binding to the FLAG peptide because TREX-2 only copurified when Sus1-FLAG was present (lanes 1 and 3). We conclude the TREX-2 complex contains at least two copies of Sus1 and the SAGA complex likely contains only one.

### Crystal Structure of the Sac3:Sus1:Cdc31 Complex

**Figure 2:**
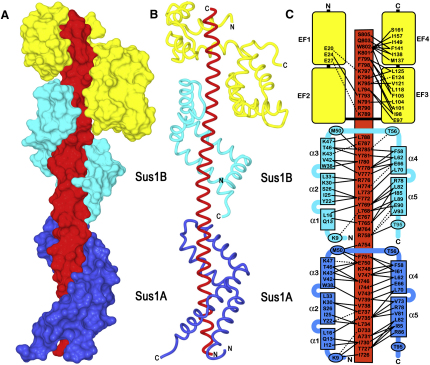
Overview of Crystal Structure of the Sac3^CID^:Cdc31:Sus1 Complex (A) Surface view. (B) Secondary structure schematic. Cdc31 is yellow, Sac3 is red, Sus1A is blue, and Sus1B is cyan. Residues 723–805 of Sac3 form a continuous, 12.5 nm-long, gently undulating, α helix to which one Cdc31 and two Sus1 (Sus1A and Sus1B) chains bind. (C) Schematic of the principal residues that are buried in the interfaces between Sac3 and its partners. Dashed lines represent putative H bonds.

The structural basis of the interactions between Sac3, Sus1, and Cdc31 was determined by crystallography. Crystals were obtained of Sac3 residues 723–805 and 753–805 complexed with both Cdc31 and Sus1. The crystals obtained with Sac3^723–805^ had a pseudotranslation in which one half of the asymmetric unit was shifted by close to half the unit cell, which makes structure solution more difficult because this effect greatly reduces the intensity of half of the reflections and so reduces the amount of data available. Therefore, we first solved the structure of the crystals formed with Sac3^753–805^. Three different crystal forms of Sac3^753–805^ complexed with full-length Cdc31 and Sus1 were obtained by vapor diffusion (Table 1). The structure of each crystal form was solved using a combination of molecular replacement and SAR (Se-Met) phasing. The model based on the 2.5 Å resolution *P2_1_* native data was refined to an R factor of 18.8% (Rfree 23.5%) with excellent geometry (Table 1) and contained Cdc31 residues 9–161, Sac3 residues 753–805 and Sus1 residues 6–95. This structure was then used to solve the Sac3^723–805^ crystals using molecular replacement and generated a 2.7 Å resolution model that refined to an R factor of 21.0% (Rfree 26.4%) with excellent geometry (Table 1). The somewhat higher values for the R factor and Rfree for this structure were a consequence of the pseudotranslation (see Chook et al., 1998). Figures 2A and 2B and Movie S1 illustrate the structure of this complex in which Sac3 was present as a long, gently undulating α helix to which were bound one Cdc31 and two Sus1 chains. The two Sus1 binding sites on Sac3^CID^ were designated Sus1A (residues 726–751) and Sus1B (residues 760–788). In the complex, Cdc31 and both Sus1 chains wrap intimately around the Sac3 helix so that the interaction interfaces involve a large number of residues. Figure 2C illustrates the principal interactions involved. Although there were extensive interfaces between Sac3 and its three partners, there was negligible interaction between Sus1A and Sus1B or between Sus1B and Cdc31.

### Cdc31:Sac3 Interaction

The fold of Cdc31 is homologous to that of calmodulin and is based on two domains, each of which contains two putative Ca-binding EF-hands. The N domain (residues 18–91) had the “closed” conformation (see Yap et al., 1999) and the C-terminal domain (residues 95–158) the “open” conformation, similar to that observed for Cdc31 bound to Sfi1 (Li et al., 2006). The Sac3:Cdc31 interaction interface (Figure 2C) involved primarily the C-terminal domain (EF hands III and IV, residues 95–158) of Cdc31, with fewer contacts observed between Sac3 and the N-terminal domain of Cdc31 than were seen with the Sfi1:Cdc31 interaction (Li et al., 2006). The residues in the Cdc31 C-terminal domain that form the large hydrophobic interface with Sac3 are similar to those involved both with the interaction with Sfi1 and Kar1 (discussed in detail in Supplemental Data). Sac3 Trp802 appeared to be central to the interaction and becomes buried in a hydrophobic cavity in the Cdc31 C-terminal domain formed by the side chains of Phe105, Met137, Ile138, Phe141, Ile149, and Ile157 (Figure 2C). The primary difference between the Cdc31 binding motifs in Sfi1 and Sac3 is the absence in Sac3 of the residues involved in the interaction with the Cdc31 N-terminal domain, and this may account for the observation of differential phenotypes in different *cdc31* mutant strains (Ivanovska and Rose, 2001).

### Sus1 Has an Articulated Helical Hairpin Fold

**Figure 3:**
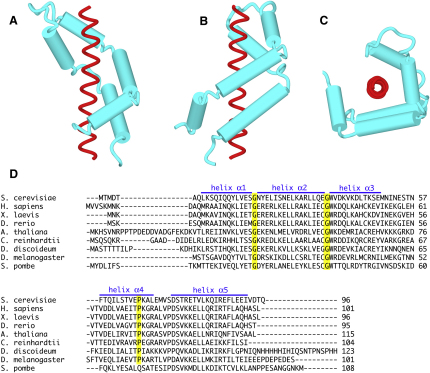
Sus1:Sac3 Interaction (A and B) The hinges between the rigid Sus1 α helices enable the molecule to wrap around the Sac3^CID^ helix, shown in red. (C) End-on view showing Sus1 wrapping around the Sac3 helix like fingers gripping a rod. (D) Sus1 sequences showing the conservation of both the helices (α1, α2, α3, α4, and α5) and the hinges between them. Single Gly residues (yellow) form the hinges between helices α1/α2 and α2/α3. A kink introduced by a Pro in helix α4 enhances the intimacy of the contact with Sac3.

The Sus1 fold was based on five α helices (helix α1 residues 8–19; helix α2 residues 21–36; helix α3 residues 38–49; helix α4 residues 58–70; and helix α5 residues 75–92), separated by flexible regions of varying size, that were arranged into an antiparallel hairpin structure that curved around the Sac3 helix (Figures 2 and 3). The curved conformation of Sus1 was facilitated by its having an articulated structure analogous to the joints in a finger with flexible hinges located between each of its five rigid α helices. The hinges enabled Sus1 to wrap around the Sac3 helix like fingers gripping a thin rod. The hinges between helices α1, α2, and α3 in the N-terminal arm of Sus1 (hinges h1 and h2) were formed from a single Gly that is conserved across species (Figure 3D), whereas the loop between the N- and C-terminal arms of the hairpin (between helices α3 and α4) contained eight residues, and that between helices α4 and α5 in the C-terminal arm (hinge h4) contained four residues. The structure of the loop between helices α3 and α4 was variable between the different Sus1 chains and was usually considerably disordered, consistent with its being highly flexible. Pro67 introduced a kink into helix α4 that helped it to wrap around the Sac3 helix. Comparison of Sus1 sequences from different species indicated that, with the exception of the heterogeneity often seen at the N and C termini of homologous proteins, this fold was strongly conserved between residues 7 and 92 (Figure 3D). The interface between the helices in the two arms of the Sus1 antiparallel hairpin was formed primarily by hydrophobic interactions and, in many ways, resembled the “knobs-in-holes” packing seen between the helices in coiled coils (Stewart and McLachlan, 1975). Putative H bonds between Glu49 and Lys68, between the Trp38 indole N-H, and the carbonyl of Ala69, and between Glu36 and both Ser74 in hinge h4 and Thr77 in helix α5 also probably contributed to the cohesion between the two arms of the hairpin. The bulky Trp38 was conserved between species, as was the small residue (Ala or Gly) at position 69 and the residues in hinge h4 that were in close contact with Trp38. The extended fold of Sus1 created a surprisingly large surface area for a protein of this size that could facilitate the interaction of Sus1 with other components of the gene expression machinery.

### Sus1:Sac3 Interaction

**Figure 4:**
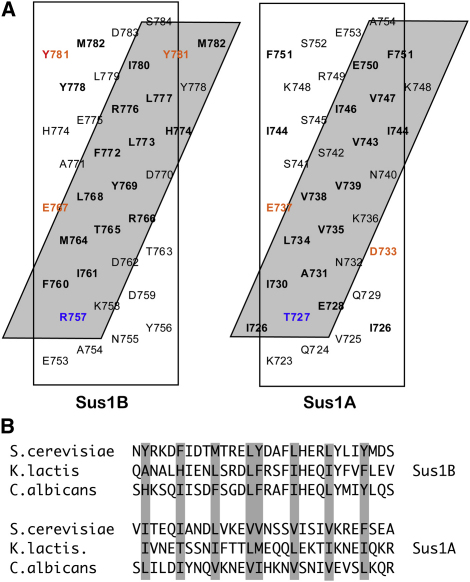
Details of the Sus1:Sac3 Interfaces (A) Hydrophobic stripe formed by the four-residue repeat in the Sac3 helix in the Sus1A and Sus1B sites. The Sac3 sequence is shown on a helical net, with residues that make a major contribution to the interface shown in bold. Residues forming putative salt bridges or H bonds are shown in red (for acidic) or blue (for basic). (B) The hydrophobic residues that give rise to the Sac3:Sus1 interface form a four-residue repeating sequence motif in which the first residue, and often the second also, are hydrophobic.

Both Sus1 hairpins coiled around the Sac3 helix in a similar way, with the Sus1A and Sus1B sites burying 1324 and 1546 Å^2^ of surface area, respectively. Almost the entire inner surface of the Sus1 hairpin interacted with Sac3 (Movie S2). Both Sus1 binding sites on Sac3 contained ∼25 residues and gave rise to a hydrophobic stripe that wound around the Sac3 helix (Figure 4A and Movie S3). This helical stripe was generated by an approximate four-residue sequence repeat motif within Sac3 (Figure 4B) in which the first two residues were hydrophobic (Phe, Tyr, Ile, Leu, or Met) or had a side chain that contained a considerable hydrophobic portion (such as Arg or Glu). Because an α helix has 3.6 residues per turn, this four-residue repeat generates a stripe that wraps around the Sac3 helix cylinder in a right-handed helix with a pitch of approximately 40 Å (Figure 4A and Movie S3). Almost the entire inner, concave surface of Sus1 was involved in the interaction with Sac3 (Movie S2), with major contributions deriving from Lys9, Tyr22, Leu29, Lys43, Lys47, Met50, Phe58, Leu82, Ile85, Arg86, Leu89, and Val93 becoming buried in the interface. The Sus1A chain appeared to have a slightly less intimate interaction with Sac3 than the Sus1B chain.

### Human Sus1/ENY2 Binds to GANP and Sac3

The presence of homologs of Sus1, Sac3, and Cdc31 in other species suggested the CID complex might be conserved. Based on the yeast CID motif structure, secondary structure predictions and sequence conservation, we identified a putative CID motif in the human Sac3/GANP (Figure S1) and showed that it binds to the human Sus1 homolog, ENY2, in vitro (Figure S4). Thus, ENY2 bound to GST-GANP(1162–1204) and GST-GANP(1162–1240) but not to the GST negative control. Additionally, ENY2 bound to GST-Sac3(723–752) and GST-Sac3(758–805) containing the Sus1A and Sus1B binding sites; however, Sus1 did not bind to either of the two GANP fragments (Figure S4).

### Sac3^CID^, Sus1, and Cdc31 Function Together for Efficient mRNA Export

To evaluate the roles of Cdc31 and each Sus1 chain we assayed mutants of the CID motif in yeast for defects in growth and mRNA export. Because previous studies on calmodulin (Montigiani et al., 1996) had shown that single point mutations in substrates were not a reliable method to alter interactions, we removed the Sus1 and Cdc31 binding sites by deleting segments of the Sac3 α helix (Figure 1C). Based on our purification and structural findings we predict the proteins bound to the CID region are as depicted in Figure 1A.

**Figure 5:**
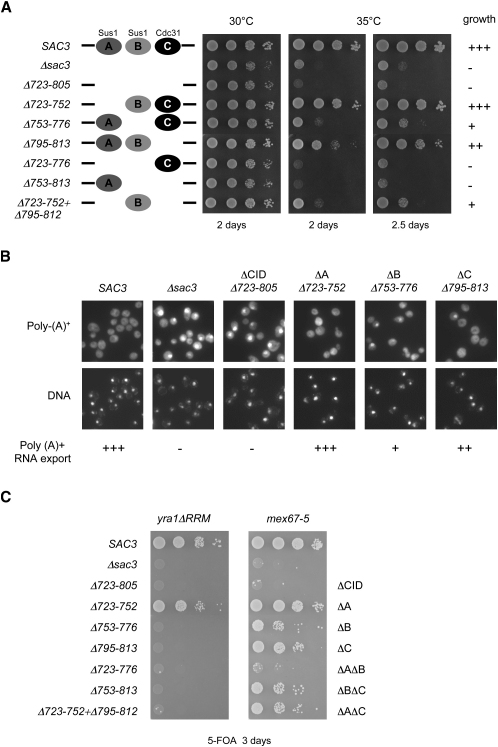
Sac3^CID^, Sus1, and Cdc31 Function Together for Efficient Growth and mRNA Export (A) Sac3 or mutant derivatives were expressed using the endogenous promoter from a single-copy plasmid (pRS315) in *sac3Δ* cells. Cells were spotted in 10-fold serial dilutions on SDC-Leu plates and growth analyzed after incubation for the indicated time and temperature. (B) *sac3Δ* cells expressing Sac3 and mutant derivatives were grown at 30°C, fixed, and poly(A)^+^ mRNA localization determined by fluorescence in situ hybridization (FISH) using a Cy3-labeled oligo-dT probe. DNA was stained with DAPI. Nuclear poly(A)^+^ mRNA retention paralleled the pattern of growth defects observed in (A), with the most pronounced accumulation seen with *sac3ΔB*, intermediate levels with *sac3ΔC*, and negligible accumulation with *sac3ΔA*. (C) Double-disrupted strains *sac3Δmex67Δ* or *sac3Δyra1Δ* harboring p*URA3*-*MEX67* or p*URA3*-*YRA1*, respectively, were transformed with the indicated plasmid-borne (pRS315-sac3 or -mex67) gene constructs. Cells were spotted in 10-fold serial dilutions on 5-FOA-containing plates and growth analyzed after incubation for 3 days at 30°C. No growth indicates synthetic lethality.

A complete deletion of *SAC3* or deletion of the CID motif (Δ723–805) results in temperature-sensitive growth. Deletion of individual binding sites for Sus1 or Cdc31 resulted in different levels of growth inhibition when assayed at 35°C (Figure 5A). The *sac3ΔB* (Δ753–776) mutant showed the strongest growth defect; the *sac3ΔC* (Δ795–813) mutant showed a lesser growth defect; and the *sac3ΔA* (Δ723–752) mutant showed no observable growth defect. These growth defects matched the pattern of poly(A)^+^ mRNA export defects observed (Figure 5B) with the most pronounced nuclear accumulation of poly(A)^+^ mRNA seen with *sac3ΔB*, intermediate levels with *sac3ΔC* and negligible accumulation with *sac3ΔA*. Significantly, deletion of any two binding sites in combination resulted in a synergistic growth defect and for *sac3ΔAΔB* (Δ723–776) and *sac3ΔBΔC* (Δ753–813) was similar to that seen with a complete deletion of the CID region (Figure 5A). Additionally, the *sac3ΔCID* mutant is synthetically lethal with mutations in the mRNA export factors Mex67 and Yra1. Notably, the combination of *sac3ΔB* (Δ753–776) or *sac3ΔC* (Δ795–813), but not *sac3ΔA* (Δ723–752) with *yra1ΔRRM* was synthetically lethal (Figure 5C). Taken together, these phenotypes indicate Sus1 and Cdc31 function together in facilitating efficient mRNA export, with Sus1 binding at the Sac3B site appearing to make a larger contribution than the binding of Cdc31 or Sus1 at the Sac3A site.

### The CID Motif Facilitates NPC Association of TREX-2

**Figure 6:**
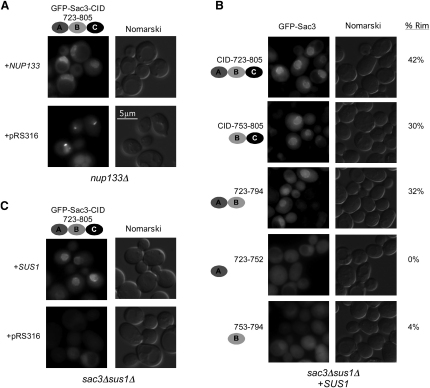
Sac3-CID(723–805) Localization to NPCs Is Dependent on Sus1 GFP-Sac3-CID fragments were expressed from plasmid with an exogenous pNOP promoter in *nup133Δ* (A) or *sac3Δsus1Δ* cells (B and C). Strains were complemented with plasmid-born *NUP133*, *SUS1*, or the corresponding empty plasmid and visualized by fluorescence microscopy and Nomarski optics. (B and C) The complete Sac3^CID^ (residues 723–805) is necessary for optimal NPC localization. Fragments of Sac3^CID^ were fused to GFP and analyzed for NPC association. The percentages of cells showing a clear GFP signal at the NPC are indicated.

Previous work showed that a GFP fusion with Sac3 residues 733–860 was located predominately at the nuclear periphery when expressed in yeast (Fischer et al., 2004). We found that expressed GFP-Sac3^723–805^ was also located mainly at the nuclear periphery (Figure 6). Moreover, when expressed in a *nup133*Δ mutant strain that causes NPC clustering, the GFP-Sac3^723–805^ signal also clustered, indicating that this fragment associates with NPCs (Figure 6A, lower panel). Further dissection of this region indicated that, as with mRNA export, there was marked synergy between the individual components. Thus, although the highest level of NPC association was seen with the complete GFP-Sac3^CID^:Sus1:Cdc31 complex, GFP-Sac3^723–794^ (that binds only Sus1A and Sus1B) and GFP-Sac3^753–805^ (that binds Sus1B and Cdc31) showed reduced but still substantial association. Strikingly, GFP fusions containing the individual Sus1A or B site (GFP-Sac3^723–752^ and GFP-Sac3^753–794^) showed very little NPC targeting (Figure 6B). Previously, the NPC localization of Sus1 was shown to require Sac3; and conversely, deletion of *SUS1* caused significant mislocalization of Sac3 (Rodriguez-Navarro et al., 2004; Köhler et al., 2008). Consistent with these observations, GFP-Sac3^723–805^ was mislocalized from the NPC when expressed in a *SUS1* deletion strain. Furthermore, the NPC localization of this fragment could be restored with coexpression of SUS1 (Figure 6C), indicating that Cdc31 alone was insufficient for NPC targeting of Sac3^CID^. Further work will be required to establish which nucleoporins are required for the NPC localization of the Sac3^CID^ complex and whether this interaction is direct or is through an additional factor.

## Discussion

The TREX-2 complex is central to the integration of transcription and mRNA nuclear export (Rodriguez-Navarro et al., 2004; Masuda et al., 2005; Aguilera, 2005), and the CID region of Sac3 and its associated binding to Sus1 and Cdc31 is crucial to this function. We have determined the crystal structure of the Sac3^CID^:Cdc31:Sus1 complex and show that Sac3^CID^ binds two Sus1 chains (Sus1A and Sus1B) and a single Cdc31 chain. Our results refine the boundaries of the CID region and indicate that Sac3 residues 723–805 are necessary and sufficient for binding both Sus1 and Cdc31. In the complex, Sac3^CID^ forms a gently undulating extended α helix, whereas Sus1 is constructed from an articulated α-helical hairpin in which five α helices are joined by flexible hinges that facilitate the molecule's wrapping around Sac3 like fingers gripping a rod. Both Sus1 and Cdc31 wrap intimately around the Sac3 helix. The crystal structure identified the precise residues in the interfaces between the CID domain and its three partner chains, and this information was employed to engineer Sac3 mutants in which the binding to each partner was compromised. Sac3 mutants in which binding to the Sus1A, Sus1B, or Cdc31 sites was ablated showed different growth and mRNA nuclear export defects. Overall, our data indicate that, within TREX-2, the Sac3^CID^ complex facilitates integration of components of the transcriptional and mRNA nuclear export functions of the gene expression pathway in yeast. Moreover, the existence of metazoan Sac3 homologs and the demonstration that the human analog GANP can bind Sus1 indicated that similar principles probably apply in metazoans.

It has previously been difficult to evaluate the precise contribution made to TREX-2 function by the interactions of Sus1 and Cdc31 with Sac3 because both Sus1 and Cdc31 also participate in other cellular functions. Thus, in addition to its role in mRNA export (Fischer et al., 2004), Cdc31 is essential for spindle pole body duplication (Ivanovska and Rose, 2001; Kilmartin, 2003) and also appears to stabilize the Kar1 kinesin (Hu and Chazin, 2003). Sus1, however, is a component of both the TREX-2 and SAGA complexes and has been proposed to provide a link between them (Rodriguez-Navarro et al., 2004). The Sac3 mutants in which the binding to each site was specifically inhibited (ΔSus1A, ΔSus1B, and ΔCdc31) were able to circumvent this difficulty and demonstrated that the binding of both Sus1 and Cdc31 to Sac3^CID^ was important for efficient mRNA nuclear export (Figure 5). Moreover, although Cdc31 and Sus1 appear to act in concert, the severity of both the growth defect and the mRNA export defect was different for each site, with the Sus1B site deletion having the largest defect and the Sus1A site deletion having the least (Figure 5). NPC localization of GFP-Sac3^CID^ fragments was only observed when Sus1B was augmented with either Sus1A or Cdc31 binding (Figure 6), again consistent with a high degree of synergy between the individual chains.

The extended α-helical conformation of Sac3^CID^ is a striking feature of the crystal structure of the Sac3^CID^:Sus1:Cdc31 complex (Figure 2). In its interactions with the spindle pole component, Sfi1 (Li et al., 2006), and the Kar1 kinesin (Hu and Chazin, 2003), Cdc31 appears to stabilize an extended α helix, analogous to the role proposed for light chains in myosin (Trybus, 2007). Isolated extended α helices are generally unstable, and so it is likely that both Cdc31 and Sus1 contribute to the function of Sac3 in the TREX-2 complex by stabilizing the 12.5 nm long, extended Sac3^CID^ α helix. Indeed, one reason two Sus1 chains are required may be that a single chain is insufficient to cover the extraordinarily long Sac3^CID^ helix. However, additional functions for the Sac3^CID^ complex are indicated by the differential effects on growth, mRNA export, and the genetic interactions with other export factors seen with deletion of the Sus1A, Sus1B, and Cdc31 binding sites of Sac3. Indeed, a major function of binding of both Sus1s to Sac3 appears to facilitate the NPC tethering of Sac3^CID^ (Figures 5 and 6) that is central to the function of the TREX-2 complex in confining actively transcribing genes to the nuclear periphery (Cabal et al., 2006; Chekanova et al., 2008). Moreover, Sus1 is thought to functionally link TREX-2 to the SAGA complex (Rodriguez-Navarro et al., 2004; Cabal et al., 2006; Köhler et al., 2008), although structural information about the interfaces involved has yet to be obtained. However, a large interface of Sus1 remains exposed after it has wrapped around Sac3, and this could bind SAGA components in the context of the complete complexes. Although further work will be required to identify the complete inventory of components that interact with the Sac3^CID^ complex, it clearly appears to function within TREX-2 to facilitate integration of interactions between the transcription and mRNA export components of the gene expression pathway in yeast.

It is presently not clear whether the binding of Sus1 and Cdc31 to Sac3^CID^ is constitutive or is regulated during the gene expression pathway. Because it is a calmodulin homolog, Cdc31 has the potential to be regulated by cellular Ca^2+^ levels. However, analogous with the results obtained with the Cdc31:Sfi1 interaction (Li et al., 2006), we were unable to see any influence of Ca^2+^ concentration on the interaction of Cdc31 with Sac3 (Figure S5). In vitro binding studies (Figure S6) indicated that Sus1 bound to Sac3 with ∼10 nM affinity, which would be consistent with its remaining constitutively attached, although this would not preclude its binding being regulated by posttranscriptional modification. There is, for example, a putative acetylation site at Sac3 Lys748 that could potentially alter the local helical conformation and thereby control Sus1 binding (Rodriguez-Navarro et al., 2004; Fischer et al., 2004).

The articulated hairpin fold of Sus1 does not appear to have close parallels in the protein structure database. However, the fold is ideally suited to wrapping around the Sac3^CID^ α helix. The flexibility introduced by the hinges between successive helices functions like the joints in a finger, enabling the relatively rigid helical segments of Sus1 to grasp Sac3. In addition, this manner of interaction leaves the outer surface of Sus1 free to bind to other components of the gene expression pathway, such as NPCs and the SAGA complex. Sus1 appears to recognize a motif characterized by hydrophobic residues repeating with an approximately four-residue period that generates a hydrophobic helical stripe that winds around the Sac3^CID^ rod. Using the information obtained on the Sus1:Sac3 interface, we were able to identify an analogous region within the human Sac3 homolog GANP and verified that it indeed bound the human Sus1 homolog DC6/ENY2. Further support for the more general applicability of the information we have obtained in yeast was recently provided by the observation that centrin 2, a protein with homology to Cdc31, was also found to be associated with NPCs and to be involved in mRNA and protein export (Resendes et al., 2008). Additionally, in *Drosophila*, Sac3/Xmas-2 and Sus1/E(y)2 are part of the AMEX complex (orthologous to yeast TREX-2) that is anchored at NPCs and regulates mRNA export (Kurshakova et al., 2007a, 2007b). These observations indicate the CID motif of Sac3 and its associated binding of Sus1 and Cdc31 is a conserved structural and functional feature in many eukaryotes.

In summary, we have determined the structure of the Sac3^CID^:Sus1:Cdc31 complex and engineered Sac3 mutants that interfere with the binding of each partner to TREX-2 to appreciate how they function in gene gating. By contributing to confining actively transcribing genes to the peripheral regions of the nucleus via its interactions with both the SAGA complex and NPCs, TREX-2 facilitates mRNA generation in the vicinity of the NPCs, increasing its local concentration at the nuclear entrance of the transport channel, which should increase the efficiency of export. Overall, our data strongly indicate that the TREX-2 Sac3^CID^ complex participates at several levels in the yeast gene expression pathway where it functions to coordinate and integrate the contributions from individual components of the machinery to promote efficient mRNA nuclear export.

## Experimental Procedures

### Construction of Yeast Strains and Plasmids

Yeast strains used in this study are listed in Table S1. Gene loci in haploid strains were C-terminally tagged or disrupted by chromosomal integration of a PCR cassette (Longtine et al., 1998). A diploid strain coexpressing Sus1-FLAG and Sus1-TAP was obtained by mating the respective haploid strains.

Plasmids pGALPATG1L-*SAC3(723–813)*; *(723–752)*; *(753–794)*; and *(733–860)* were created by PCR amplification of the designated residues and subsequent cloning into the NcoI and BamHI sites of pGALPATG1L (Künzler et al., 2000). pGALPATG1L*-SAC3(795–813)* was created by annealing oligonucleotides coding for residues 795*–*813 and cloning into NcoI and BamHI. pNOPGFP-SAC3 fragment constructs were created by PCR amplification (723*–*805) and (723*–*794) or subcloning (723*–*752) and (753*–*794) from corresponding pGALPATG1L-SAC3 constructs and cloning into the PstI and SalI or XhoI sites of pNOPGFPA1L (Hellmuth et al., 1998) and contain a TEV site between the GFP and Sac3 fragment. *SAC3* deletions were created by PCR amplification and cloning of the resulting fragment into the PmeI and EagI sites of pNOPATAIL-*SAC3* or pRS315-*SAC3* (Fischer et al., 2002). *Sac3Δ795–813 and sac3Δ753–813* deletions contain an added NcoI site after residue 813 and *sac3Δ723–752* + *Δ795–812* contains an AatII site before residue 723 and a SnaBI site after residue 812.

### Protein Expression and Purification

Sus1 cDNA was cloned into untagged expression vector pOPT (Teo et al., 2006), whereas Cdc31 was cloned into pET30a (Novagen; Beeston, UK). Sac3 fragments were generated by PCR from synthetic Sac3 cDNA spanning residues 720–860, codon optimized for expression in *E. coli* (see Supplemental Data), and cloned into pGEXTEV (Matsuura and Stewart, 2004), a modified version of pGEX-4T-1 (GE Healthcare; Chalfont St. Giles, UK) in which the thrombin site has been replaced by a TEV protease site. A synthetic GANP fragment corresponding to residues 1162–1462 was constructed (Eurofins MWG; Ebersberg, Germany) and used as a template for PCR to create and clone smaller GANP fragments into pGEXTEV. ENY2 cDNA was cloned into the NdeI and SalI sites of pET28a (Novagen).

Sus1 was expressed in BL21(DE3) CodonPlus RIL cells in ZYM-5052 auto-inducing medium (Studier, 2005) at 20°C. GST-Sac3 fragments and Cdc31 were coexpressed in the same way. All protein purification steps were performed at 4°C, unless stated otherwise. Cells were lysed by high-pressure cavitation (10–15k psi) in 50 mM Tris-HCl (pH 8.0), 25% w/v sucrose, 1 mM ethylene glycol tetraacetic acid (EGTA), and 1 mM phenylmethanesulphonylfluoride (PMSF). Clarified cell lysate, containing the GST-Sac3 fragment and untagged Cdc31, was mixed with clarified lysate containing untagged Sus1 to form the complex which was then bound to glutathione Sepharose 4B resin (GE Healthcare) for 1 hr. The resin was washed with 500 ml of 50 mM Tris-HCl (pH 8.0), 200 mM NaCl, 1 mM EGTA, 1 mM DTT to remove excess unbound Sus1 and Cdc31, and the Sac3:Cdc31:Sus1 complex then released by overnight digestion at room temperature with 100 μg of His-TEV protease (S219V mutant; Kapust et al., 2001). The complex was purified on a HiLoad Superdex 75 26/60 prep-grade column (GE Healthcare) in 20 mM Tris-HCl (pH 8.0), 50 mM NaCl, 1 mM ethylenediaminetetraacetic acid (EDTA), and 1 mM DTT. SeMet-labeled protein was expressed by conventional isopropyl-beta-D-thiogalactopyranoside (IPTG) induction in B834(DE3) cells as described (Teo et al., 2006) and complexes formed and purified as above, but in the presence of 5 mM DTT.

GANP fragments and ENY2 were expressed individually in BL21 cells. Cultures were grown at 37°C to an OD_600_ = 0.5, then induced by addition of IPTG to 0.5 mM and grown at 23°C for 3 hr. Cells were lysed in NB buffer (150 mM NaCl, 50 mM KOAc, 20 mM Tris, 2 mM Mg(OAc)_2_ and 0.1% NP-40) containing 5 mM DTT. GANP fragments were bound to glutathione Sepharose 4B resin for 1 hr at 4°C and beads washed with NB buffer to remove unbound proteins. 6XHis-ENY2 and 6XHis-Sus1 were purified over Ni-NTA Agarose (QIAGEN; Crawley, UK) and eluted with 150 mM imidizole.

Protein A-tagged Sac3 fragments were purified from pellets from 2 l of yeast cell culture grown in SRC-Leu at 30°C until OD_600_ = 1.0, at which time 5% galactose was added and growth was continued for 5 hr. Protein A/GST and protein A/Flag split-purifications used pellets from 2 l and 4 l, respectively, of yeast cell culture (OD_600_ = 3.5) grown in YPD at 30°C. Lysis and purification of Protein-A-tagged proteins were performed in LB buffer containing 50 mM Tris-HCl (pH 7.5), 100 mM NaCl, 1.5 mM MgCl_2_, 0.15% NP-40, and 0.5 mM DTT essentially as described (Puig et al., 2001). IgG-bound Protein A Sac3 fragments were washed once with 50 mM Tris (pH 7.6), 150 mM NaCl, 0.05% Tween 20 and once with 5 mM NH_4_Ac (pH 5.0) before elution with 0.5 M HAc (pH 3.4). In split-tag purifications, the TEV-eluted protein samples were subsequently incubated with glutathione Sepharose or anti-FLAG M2 affinity resin. GSH bound protein was washed with LB buffer and eluted with 10 mM glutathione, and anti-FLAG bound protein was washed with LB containing 200 mM NaCl and eluted with free FLAG peptide. When required, samples were then separated on NuPAGE SDS 12% or 4%–12% gradient gels run in MES or SDS-PAGE buffer (Invitrogen; Paisley, UK) and stained with colloidal Coomassie (Sigma; Dorset, UK). Mass spectrometric identification of the proteins contained in Coomassie-stained bands was performed as described (Nissan et al., 2002).

### Crystallization and Data Collection

Crystals were grown in PEG solutions (Table 1) at 19°C by hanging drop vapor diffusion using microseeding to optimize single crystal growth. When required, crystals were exposed to cryoprotectant briefly before flash cooling in liquid nitrogen prior to data collection. Crystal form 3 was dehydrated for 12 hr in solution similar to mother liquor, but containing 21% w/v PEG4K, prior to freezing. Crystallographic data were collected at the European Synchrotron Radiation Facility (Grenoble, France) (Table 1).

### Structure Solution and Refinement

The structure of three crystal forms of the Sac3^753–805^:Cdc31:Sus1 complex was determined by a combination of molecular replacement, using the structure of Cdc31 (Li et al., 2006) and SAD phasing as described in detail in Supplemental Data. After iterative cycles of refinement and rebuilding, the 2.5 Å resolution *P2_1_* data set resulted in a model with an R factor of 18.8% (Rfree = 23.5%) and excellent geometry (Table 1). This structure was then used to obtain the structure of the Sac3^723–805^ complex by molecular replacement followed by building an additional Sus1 chain and Sac3 residues 723*–*755 for each of the four copies of the complex in the *P2_1_* asymmetric unit as described in detail in Supplemental Data. The final 2.7 Å resolution model for the Sac3^723–805^ complex had an R factor of 21.0% (Rfree 26.4%) and excellent geometry (Table 1).

### In Vitro Binding Assays

Binding assays were performed by immobilizing GST fusion protein containing complexes on glutathione Sepharose 4B resin from clarified bacterial cell lysate(s). Cell lysates coexpressing GST-Sac3 fragments and untagged Cdc31 were mixed with cell lysate expressing untagged Sus1 and incubated with resin at 4°C for 1 hr. The resin was washed with 50 mM Tris-HCl (pH 8.0), 200 mM NaCl, 1 mM EGTA, and 1 mM DTT. Purified ENY2 was added in 10-fold excess to equal amounts of immobilized GST or GST-GANP fusions and washed extensively with NB buffer. Samples were analyzed by SDS-PAGE.

### In Situ Hybridization and Fluorescence Microscopy

Cells expressing GFP fusions were grown in selective media at 23°C to logarithmic phase. Fluorescence microscopy was performed as described (Grund et al., 2008). In situ hybridization of poly(A)^+^ RNA was performed essentially as described previously (Amberg et al., 1992) except that the primer was labeled with Cy3. Approximately 350 cells were analyzed to determine the percentage with observable nuclear rim staining.

## Figures and Tables

**Table 1:** Crystal Data

Crystals			
Sac3 fragment	753–805	753–805	723–805
Crystal form	1	2	3
Composition	native	Cdc31, Sac3, SeMet	native
Crystallization conditions	100 mM MES (pH 6.5), 16% w/v PEG4K, 20% w/v glycerol	100 mM Bis-tris (pH 6.5), 24% w/v PEG3350,	16% w/v PEG4K, 20% w/v glycerol, 0.2 M (NH_4_)_2_SO_4_
Cryoprotectant	none required	15% w/v PEG400	dehydrated
Space group	*P2_1_*	*P2_1_*	*P2_1_*
Unit cell dimensions			
a (Å)	53.5	47.3	73.6
b (Å)	60.1	115.2	122.7
c (Å)	77.3	61.4	128.7
β (°)	108.7	91.0	95.75

Data Collection			

ESRF beamline	ID23	ID29	ID14-1
Wavelength (Å)	0.979	0.979	0.979
Resolution range (Å)∗	28–2.5 (2.64–2.5)	20–2.8 (2.95–2.8)	20–2.7 (2.85–2.7)
Total observations∗	53,086 (7700)	52,734 (6391)	670,858 (67,275)
Unique observations∗	16,233 (2339)	14,914 (2075)	61,904 (9047)
Completeness (%)∗	99.7 (99.8)	92.0 (88.1)	99.1 (99.1)
Multiplicity	3.3 (3.3)	3.5 (3.1)	10.8 (7.4)
R_merge_ (%)∗	6.9 (43.1)	8.7 (37.9)	11.4 (78.2)
Mean I/σ(I)∗	9.7 (2.7)	7.5 (2.1)	14.7 (2.8)

Refinement			

R_cryst_/R_free_ (%)	18.8/23.5	19.3/24.8	21.0/26.4
Bond length rmsd (Å)	0.012	0.012	0.006
Bond angle rmsd (°)	1.4	1.3	0.9
Ramachandran plot (%)			
Core region	95.3	93.8	94.5
Allowed	4.7	6.2	5.5
Generously allowed	0	0	0
Forbidden	0	0	0

∗ Parentheses refer to final resolution shell.

## Accession Numbers

The coordinates and structure factors for the 2.5 Å resolution Sac3^753–805^ and the 2.7 Å resolution Sac3^723–805^ complexes have been deposited in the Protein Data Bank with ID codes 3FWB and 3FWC, respectively.

## Supplemental Data

Document S1. Supplemental Text and Six Figures

Movie S1. The Sac3^CID^:Cdc31:Sus1 Complex Sac3 is red; Cdc31 is yellow; Sus1A is blue; Sus1B is cyan.

Movie S2. Sus1 Interaction Interface with Sac3^CID^ Most of the inner surface of Sus1 (dark blue) is in contact with the Sac3^CID^ helix (red). The movie illustrates the contact between Sus1B and residues 753–790 of Sac3.

Movie S3. Sac3^CID^ Interface with Sus1 Surface view of the Sac3 helix (residues 753–790; red) showing how the residues that form the interaction interface with Sus1 (Sus1B, cyan) form a helical stripe (dark red) that winds around the Sac3 α helix.
