# Supplementary material for: Sus1, Cdc31, and the Sac3 CID Region Form a Conserved Interaction Platform that Promotes Nuclear Pore Association and mRNA Export
Source: Mol Cell. 2009 Mar 27;33(6-2):727–37. doi: 10.1016/j.molcel.2009.01.033 (PMC2726291; doi:10.1016/j.molcel.2009.01.033)

Molecular Cell, Volume *33*

Supplemental Data

Sus1, Cdc31, and the Sac3 CID Region Form a Conserved Interaction Platform that Promotes Nuclear Pore Association and mRNA Export

Divyang Jani, Sheila Lutz, Neil J. Marshall, Tamás Fischer, Alwin Köhler, Andrew M. Ellisdon, Ed Hurt, and Murray Stewart

Codon-optimised Sac3 720-860 sequence.

A synthetic Sac3 fragment spanning residues 720-860, codon-optimized for expression in *E. coli*, was constructed using a modification of Young and Dong (2004). Sequential pairs of phosphorylated primers with complementary overhangs corresponding to the codon-optimized Sac3$^{720\text{-}860}$ coding sequence were annealed and ligated to generate a series of short, overlapping DNA fragments. The full-length Sac3$^{720\text{-}860}$ codon-optimized sequence was obtained by using conventional PCR with two outer primers and the series of overlapping DNA fragments as DNA templates.

```
AACGCGGATAAACAGGTGATTACCGAACAGATTGCGAACGATCTGGTGAAAGAAGTGGTGAACAGCAGCG
TGATCAGCATCGTGAAACGTGAATTCAGCGAAGCAAACTACCGTAAAGATTTCATCGATACCATGACCCG
TGAACTGTACGATGCATTCCTGCATGAACGTCTGTACCTGATCTACATGGATAGCCGTGCAGAACTGAAA
CGTAACAGCACCCTGAAAAAAAAATTCTTCGAAAAATGGCAGGCAAGCTACAGCCAGGCAAAAAAAAACC
GTATCCTGGAAGAAAAAAAACGTGAAGAAATCAAACTGGTGAGCCATCAGCTGGGTGTGCCGGGTTTCAA
AAAAAGCACCTGCCTGTTCCGTACCCCGTACAAAGGTAACGTGAACAGCAGCTTCATGCTGAGCAGCAGC
GAT
```

Identification of a minimal region of Sac3^{CID}, containing binding sites for both Sus1 and Cdc31, allowed preparation of high-resolution crystals.

Previous work established that the CID domain of Sac3, that encompassed residues 733-860, binds both Cdc31 and Sus1 (Fischer et al. 2004). Comparison of the Sac3 CID domain sequence with other Cdc31-binding peptides suggested that a putative Cdc31 binding site was probably located between residues 795 and 813. This sequence (KKKFFEKWQASYSQAKKNR) has a number of characteristics in common with other Cdc31 binding motifs (see Li et al. 2006; Kilmartin 2003). Thus, it starts with positively charged amino acids (KKK), followed by a negatively charged amino acid (E) and a bulky hydrophobe (W), followed by hydrophobic amino acids (A and Y) and ending in a group of charged amino acids (KKR) (Fischer et al. 2004). Consistently, in yeast, residues 795-813 of Sac3 co-purified with Cdc31 (**Figure 1B**) and deletion of the same residues resulted in loss of Cdc31 binding but did not alter Sus1 binding to

Sac3 (**Figure 1C**). Secondary structure prediction indicated that Sac3 residues 723-830 probably formed an α-helix, similar to that of Sfi1 in the Sfi1:Cdc31 complex (Li et al. 2006).

Initial crystals of complex containing Sac3 residues 753-813 diffracted very poorly. Therefore, truncation mutants were used to define more precisely the region of Sac3 that binds Sus1 and Cdc31. A series of Sac3 fragments were made that progressively removed residues from the C-terminus of the centrin-binding site. In this way, a fragment comprising residues 753-805 was found that retained the ability to bind both Sus1 and Cdc31 (**Figure S3**). Crystals grown from this minimal Sac3 fragment yielded high-resolution diffraction data. A Sac3 construct spanning residues 723-805, incorporating both Sus1 binding sites and also ending in residue 805, yielded high resolution data readily.

Crystal structure determination

An initial MR solution of the $P2_1$ crystal of the $Sac3^{753\text{-}805}$:Sus1:Cdc31 complex (crystal form 2, **Table 1**) was obtained using Cdc31 (PDB 2GV5) as model. Although the phasing was not adequate to trace the Sac3 and Sus1 chains reliably, it enabled identification of the Se sites present in Sac3 (2 sites) and Cdc31 (4 sites). The resultant SAD map obtained after processing using SHARP (Bricogne et al. 2003) followed by solvent flipping (Abrahams & Leslie, 1996) enabled most of the Sac3 chain to be built, after which solvent-flattened maps began to show a series of α-helices from which a putative Sus1 model was built. Molecular replacement was then used to place this model into $P2_12_12_1$ crystals formed from unlabelled Sac3 and Cdc31 and Se-Met labelled Sus1. Identification of the two Se sites on the Sus1 chain allowed unequivocal assignment of the Sus1 sequence and some rebuilding of the Sus1 chain. This structure was finally placed into the 2.5Å resolution $P2_1$ native data set (crystal form 1, **Table 1**) and, after iterative cycles of refinement (using REFMAC5 with TLS (CCP4, 1994) and CNS (Brunger, 2007) together with local rebuilding, resulted in a model with an R-factor of 18.8% (Rfree = 23.5%) and excellent geometry (**Table 1**). This structure had a MolProbity (Davis et al. 2007) score of 1,85 (97th percentile). We also obtained the 3.1 Å resolution molecular replacement structure (R-factor=19.9%; Rfree=24.6%) of the $P2_12_12_1$ crystal form that showed the same arrangement of chains in the complex.

The structure of the $Sac3^{753\text{-}805}$ complex was then used to obtain the structure of the $Sac3^{723\text{-}805}$ complex by molecular replacement using MOLREP (CCP4, 1994). These crystals diffracted to 2.7 Å resolution (**Table 1**), although the diffraction data showed some asymmetry, which was corrected using the anisoscale web site (http://www.doe-mbi.ucla.edu/~sawaya/anisoscale). The self-Patterson function showed that there was a clear pseudo-translation of (0.5, 0, 0.5) which complicated the determination, although eventually a model that contained four copies of the $Sac3^{753\text{-}805}$ complex was obtained. Sac3 residues 723-752 and an additional Sus1 chain were then built into the density and the structure refined, initially using CNS (Brunger, 2007) followed by REFMAC (CCP4, 1994) and finally PHENIX (Terwilliger et al. 2008) using TLS refinement using the groups indicated by the TLSMD website (http://www.skuld.bmsc.washington.edu/~tlsmd/index.html - Painter and Merritt, 2007) to yield a final structure that contained four Cdc31 chains, four Sac3 chains, eight Sus1 chains, 4 sulphate ions, and 108 water molecules and which had an R-factor of 21.0% (Rfree = 26.4%) and excellent geometry (**Table 1**). This structure had a MolProbity (Davis et al. 2007) score of 2.13 (92nd percentile).

The Cdc31:Sac3 interface

The fold of Cdc31 is homologous to that of calmodulin and is based on two domains, each of which contains two putative Ca-binding EF-hands. The N-domain (residues 18-91) had the "closed" conformation (see Yap et al. 1999) and the C-terminal domain (residues 95-158) in the "open" conformation, similar to that observed for Cdc31 bound to Sfi1 (Li et al. 2006). As observed in Cdc31:Sfi1 crystals (Li et al. 2006), there was little clear electron density for Cdc31 residues 1-13 in any of the different crystal forms we investigated. These residues vary between species and are not required for function (Li et al. 2006) and were probably disordered. The Sac3:Cdc31 interaction interface involved primarily the C-terminal domain (EF hands III and IV, residues 95-158) of Cdc31, with fewer contacts observed between Sac3 and the N-terminal domain of Cdc31 than were seen with the Sfi1:Cdc31 interaction (Li et al. 2006). The residues in the C-terminal domain of Cdc31 that form the large hydrophobic interface with Sac3 are similar to those involved both with the interaction with Sfi1 (Li et al. 2006) and the analogous interaction between *Clamydomonas* centrin and Kar1 (Hu & Chazin, 2003), albeit the Sac3 helix is oriented in the opposite direction. Trp802 of Sac3 appears to play a central role in the interaction and becomes buried in a hydrophobic cavity in the C-terminal domain of Cdc31 formed by the side chains of Phe105, Met137, Ile138, Phe141, Ile149 and Ile157 (**Figure 2C**). Additional hydrophobic contacts are also made by Phe799^{Sac3} and Phe798^{Sac3} that contact smaller hydrophobic patches on Cdc31 formed by Phe105, Leu118, Leu125 and Glu97, Ile98, Ala101, respectively. There are also putative salt bridges formed between Lys795^{Sac3} - Glu124^{Cdc31} and Arg790^{Sac3} - Glu97^{Cdc31}. By comparison, the interaction interface between Sac3 and the Cdc31 N-terminal domain is much less substantial and comprises hydrophobic contacts between Met782^{Sac3} - Val47^{Cdc31} and Thr793^{Sac3} - Glu24^{Cdc31} together with putative salt bridges between Lys789^{Sac3} - Glu27^{Cdc31} and Lys797^{Sac3} - Glu20^{Cdc31}. The ~24-residue Cdc31-binding region of Sac3 is somewhat shorter than the ~33 residue Cdc31-binding motifs present in Sfi1 (Li et al. 2006). Although the Cdc31 binding region of Sac3 is not directly homologous to the consensus Cdc31 binding motif seen in Sfi1 (Li et al. 2006), it clearly shares several common features, especially the roles played by Phe798, Phe799 and Trp802 that dominate the interaction interface with the Cdc31 C-terminal domain. The primary difference between the Cdc31 binding motifs in Sfi1 and Sac3 is the absence in Sac3 of the residues involved in the interaction with the Cdc31 N-terminal domain.

Affinity of Sus1 for Sac3^{CID}

To measure the binding affinity of S-tagged Sus1 to the Sac3:Cdc31 complex Fluotrac-600 96-well black plates (Greiner Bio-one) or 96-well black glutathione coated plates (Pierce) were coated with 1 to 5 nM per well of GST-Sac3$^{723\text{-}805}$:Cdc31 complex or GST-alone for 16 hours at room temperature. Solid phase binding assays were then performed as described (Bayliss et al. 2002), except that binding was carried out for 16 hours at 4 °C. The bound S-tagged Sus1 was detected by incubation with horseradish peroxidase conjugated S-tag antibody (Abcam, Cambridge, UK) and subsequent addition of the horseradish peroxidase substrate SuperRed (Virolabs, Virginia, USA). The fluorescent signal was determined using a Tecan Safire II plate reader at excitation and emission wavelengths of 530 nm and 590 nm respectively. Binding data (**Figure S6**) were analyzed with GraphPad Prism 5.0a for Mac OS X (GraphPad Software, San Diego, California, USA) using nonlinear regression assuming one site binding.

Supplemental References

Abrahams, J.P. and Leslie, A.G. (1996). Methods used in the structure determination of bovine mitochondrial F1 ATPase. Acta Crystallogr. *D52*, 30-42.

Bayliss, R., Littlewood, T., Strawn, L.A., Wente, S.R. and Stewart, M. (2002). GLFG and FxFG nucleoporins bind to overlapping sites on importin-β. J. Biol. Chem**.** *277*, 50597-50606.

Bricogne, G., Vonrhein, C., Flensburg, C., Schiltz, M. and Paciorek, W. (2003). Generation, representation and flow of phase information in structure determination: recent developments in and around SHARP 2.0. Acta Crystallogr D Biol Crystallogr. 59, 2023-2030.

Brunger, A.T. (2007). Version 1.2 of the Crystallography and NMR system. Nature Protoc. *2*, 2728-2733.

CCP4 (1994). The CCP4 suite: programs for protein crystallography. Acta Crystallogr. *D50*, 760-763.

Davis, I.W., Leaver-Fay, A., Chen, V.B., Block, J.N., Kapral, G.J., Wang, X., Murray, L.W., Arendall, W.B., Snoeyink, J., Richardson, J.S. and Richardson, D.C. (2007). MolProbity: all-atom contacts and structure validation for proteins and nucleic acids. Nucleic Acids Res. 35, W375-W383.

Painter, J. and Merritt, E.A. (2006). Optimal description of a protein structure in terms of multiple groups undergoing TLS motion. Acta Crystallogr *D62*, 439-450.

Terwilliger, T.C., Grosse-Kunstleve, R.W., Afonine, P.V., Moriarty, N.W., Zwart, P.H., Hung, L.W., Read ,R.J. and Adams, P.D. (2008). Iterative model building, structure refinement and density modification with the PHENIX AutoBuild wizard. Acta Crystallogr D Biol Crystallogr. *64*, 61-69.

Young, L. and Dong, Q. (2004). Two-step total gene synthesis method. Nucleic Acids Res. *32*, e59.

Table S1. Yeast strains used in this study

Strain	Genotype	Reference
sac3ΔG1	*Mat a ; ade2, his3, leu2, trp1, ura3, sac3::kanMX4*	(Fischer et al., 2002)
sac3Δ THP1-GST SUS1-MYC	*Mat a ; ade2, his3, leu2, trp1, ura3, sac3::kanMX4, THP1::HIS3, SUS1-MYC::TRP1*	This study
SUS1-TAP	*Mat a ; ade2, his3, leu2, trp1, ura3, SUS1-TAP::TRP1*	(Rodriguez-Navarro et al., 2004)
SUS1-TAP/*SUS1*-FLAG	*Mat a/a ; ade2/ade2, his3/his3, leu2/leu2, trp1/trp1, ura3/ura3, SUS1-TAP::TRP1/SUS1-FLAG::TRP1*	This study
sac3ΔYRA1 shuffle	*Mat α ; ade2, his3, leu2, trp1, ura3, sac3::kanMX4, yra1::HIS3*	(Fischer et al., 2002)
sac3Δ MEX67 shuffle	*Mat α ; ade2, his3, leu2, trp1, ura3, sac3::kanMX4, mex67::HIS3*	(Fischer et al., 2002)
sac3Δ sus1Δ	*Mat α ; his3, leu2, ura3, sus1::KanMX4, sac3::NAT*	This study
nup133Δ	*Mat a ; ade2, his3, leu2, trp1, ura3, nup133::HIS3*	(Doye et al., 1994)

Figure S1. Sequence alignment of the Sac3 CID region with homologous proteins. Sac3 homologs were identified by BLAST searches. Sequences downstream of the conserved PFAM Sac3/GANP domain were submitted to Jpred (http://www.compbio.dundee.ac.uk/~www-jpred) for secondary structure prediction. Regions predicted to form long α-helices were then further aligned using ClustalW2 (http://www.ebi.ac.uk/Tools/clustalw2). Amino acids are coloured based on chemical properties and aligned conservation. Numbers above the alignment correspond to human GANP and below to yeast Sac3 amino acid residues.

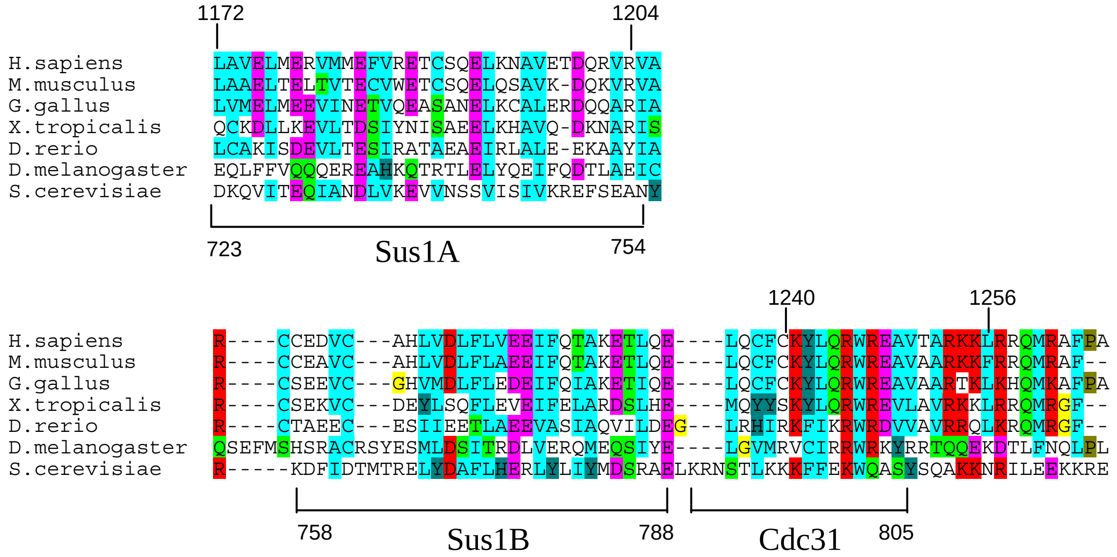

Figure S2. Over expressed Sac3 sequences which bind Cdc31 are toxic to yeast. Sac3 sequences under the control of the *GAL* promoter were expressed in *sac3Δ* cells and spotted in 10-fold serial dilutions on glucose (SDC-Leu) or galactose (SGC-Leu) containing plates and growth was analyzed after incubation for 3 days at 30°C.

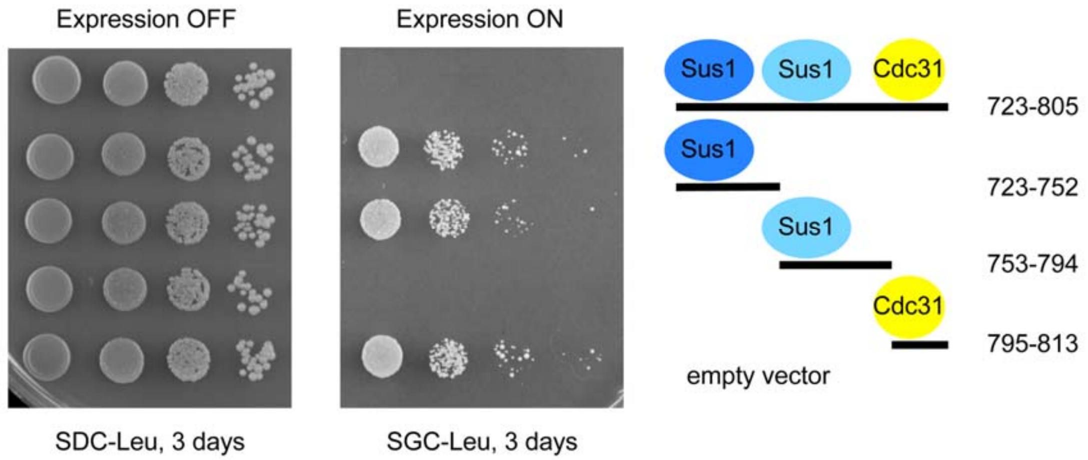

Figure S3. A minimal fragment of the Sac3^{CID} (residues 753-805) retains binding to Sus1 and Cdc31. Co-expressed GST-Sac3 fragments and Cdc31 together with singly expressed Sus1, both from bacterial cell lysates, were incubated with glutathione sepharose for 1hr, the resin washed thoroughly and analysed by SDS-PAGE.

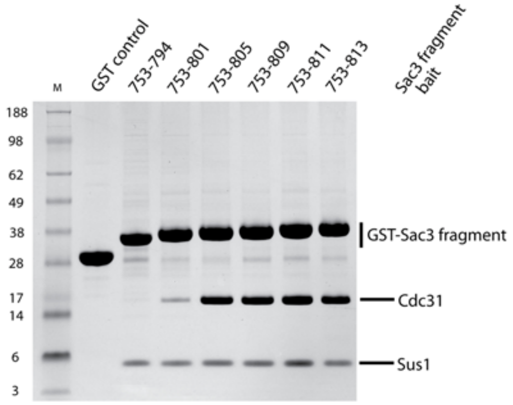

Figure S4. Human Sac3/GANP binds human Sus1/ENY2 *in vitro*. GST-Sac3(723-752) contains the Sus1A site whereas GST-Sac3(758-805) contains the Sus1B and Cdc31 sites. GST-GANP(1162-1204) contains the putative Sus1A site, whereas GANP(1162-1240) contains both the putative binding Sus1 sites identified by sequence analysis (**Figure S1**). Recombinant fragments, GST-GANP or GST-Sac3, or GST alone were immobilized on glutathione sepharose and incubated with purified recombinant His-tagged ENY2 or His-tagged Sus1. The sepharose beads were washed to remove non-specifically bound protein and analyzed by SDS-PAGE and Coomassie staining.

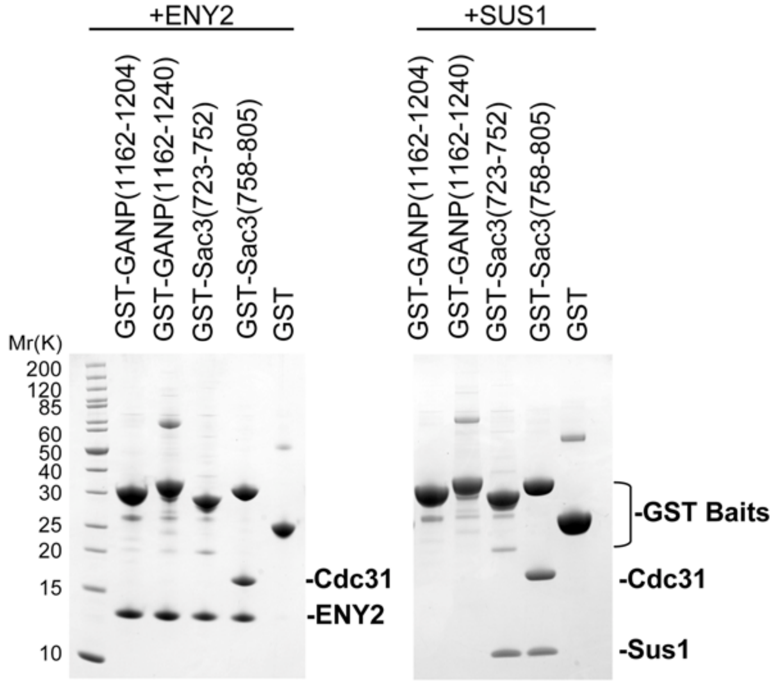

Figure S5. Sac3^{CID}:Cdc31:Sus1 complex formation is not influenced by calcium availability. The complex between GST-tagged Sac3$^{723-805}$, Cdc31 and Sus1 was immobilised on glutathione sepharose from clarified bacterial lysate in the presence of 5mM EGTA or 5mM $CaCl_2$. After 1hr incubation, resins were thoroughly washed in buffer containing either 5mM EGTA or 5mM $CaCl_2$ and analysed by SDS-PAGE.

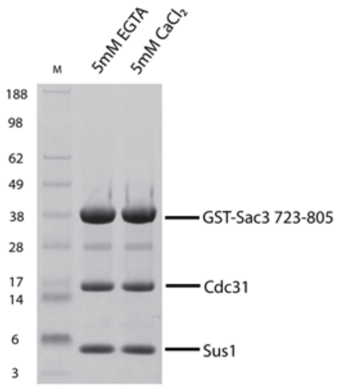

Figure S6. Affinity of Sus1 for Sac3^{CID}:Cdc31. The affinity of Sus1 for the Sac3^{CID}:Cdc31 complex was measured using solid phase binding assays (Bayliss et al. 2002). The apparent dissociation constant was calculated from the average of ten normalized data sets using non-linear regression and is expressed ± the standard error.

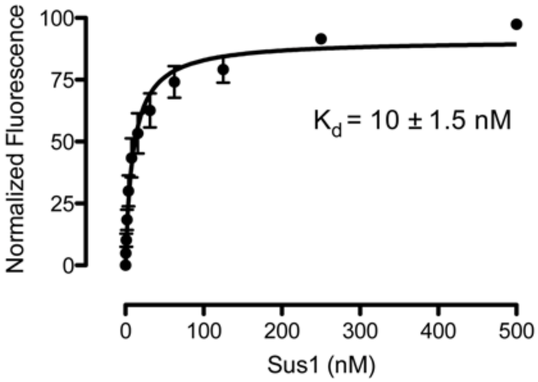

Supplement: Document S1. Supplemental Text and Six Figures [file mmc1.pdf]
