# Supplementary figures and images for: Sus1, Cdc31, and the Sac3 CID Region Form a Conserved Interaction Platform that Promotes Nuclear Pore Association and mRNA Export
Source: Mol Cell. 2009 Mar 27;33(6-2):727–37. doi: 10.1016/j.molcel.2009.01.033 (PMC2726291; doi:10.1016/j.molcel.2009.01.033)

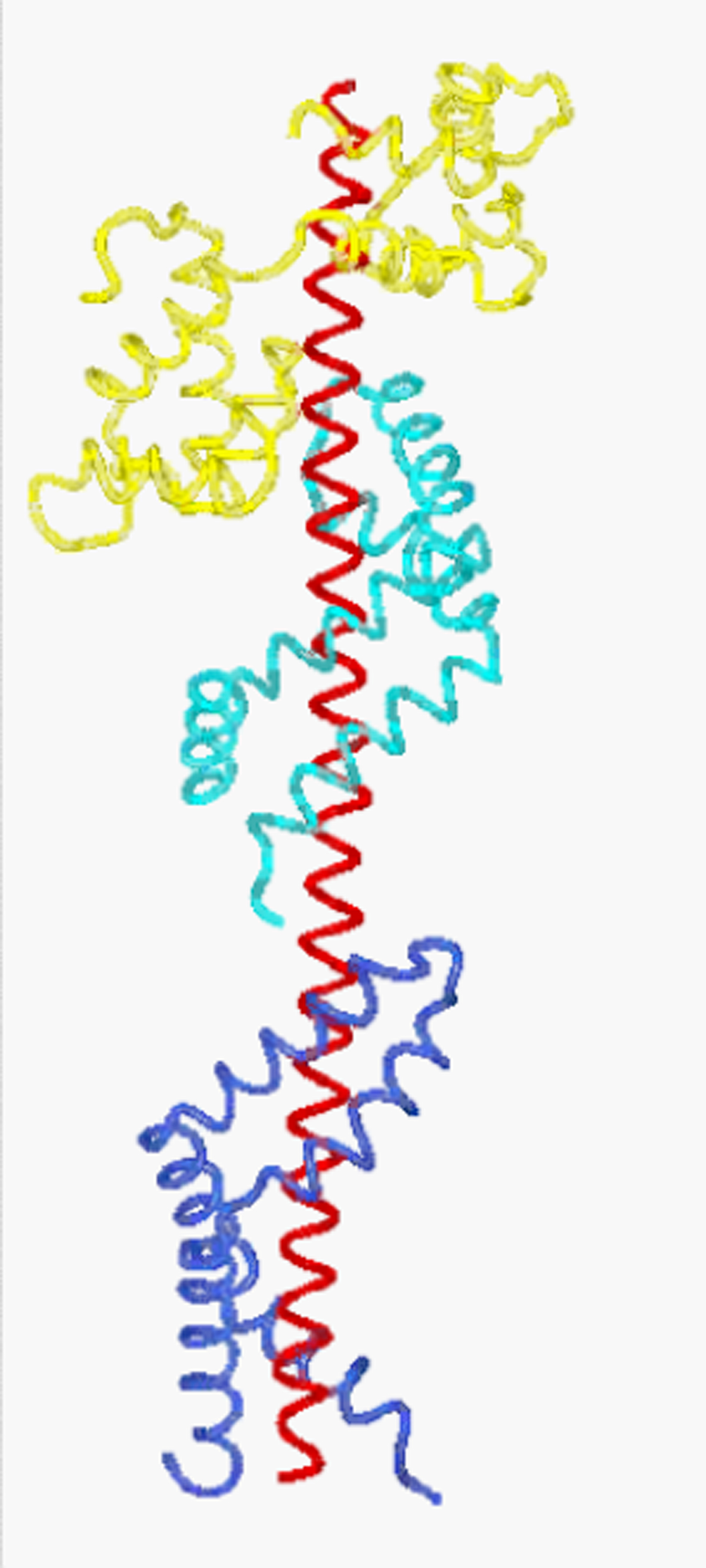

Supplement: Movie S1. The Sac3CID:Cdc31:Sus1 Complex — Sac3 is red; Cdc31 is yellow; Sus1A is blue; Sus1B is cyan. [file mmc2.jpg]

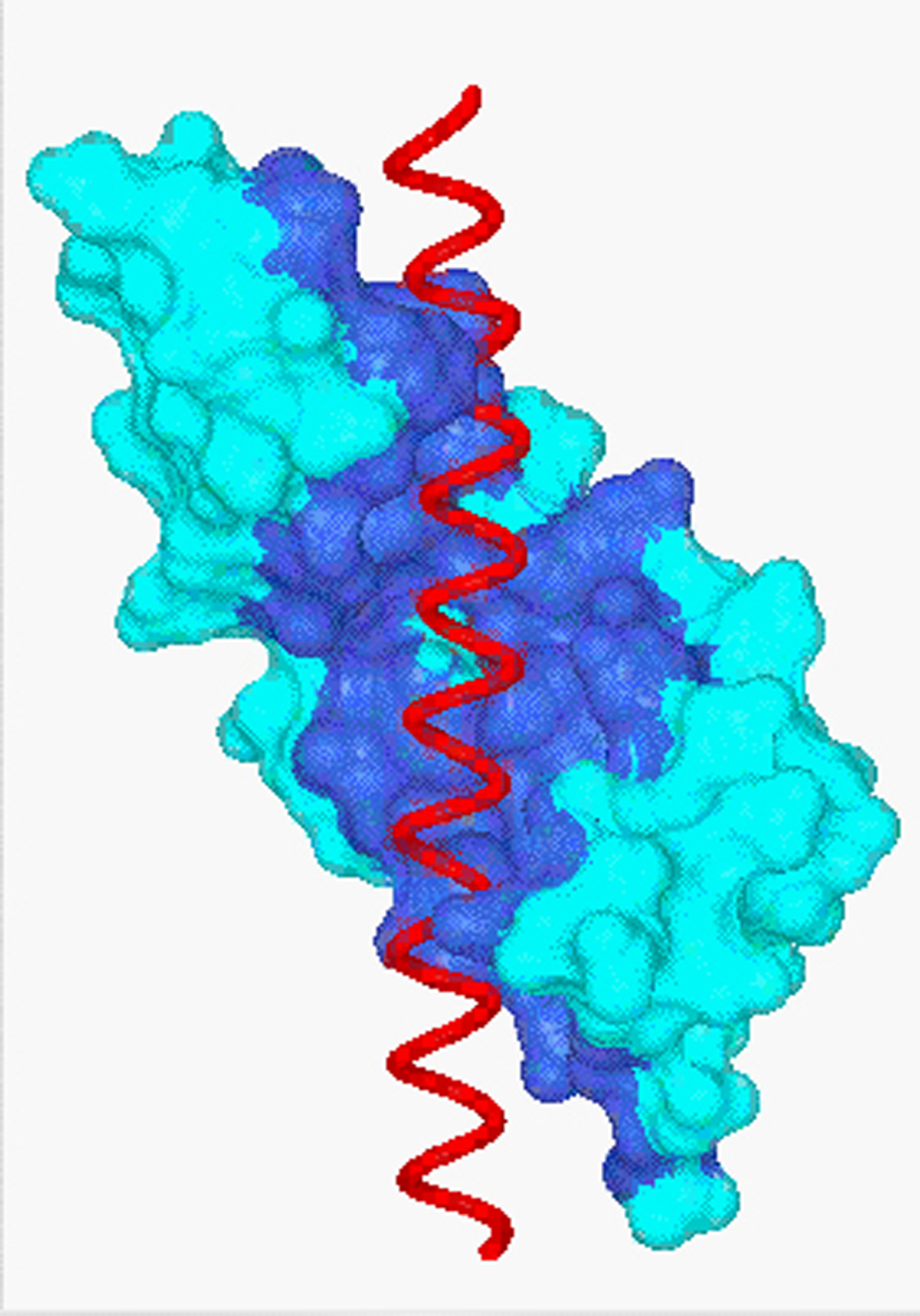

Supplement: Movie S2. Sus1 Interaction Interface with Sac3CID — Most of the inner surface of Sus1 (dark blue) is in contact with the Sac3CID helix (red). The movie illustrates the contact between Sus1B and residues 753–790 of Sac3. [file mmc3.jpg]

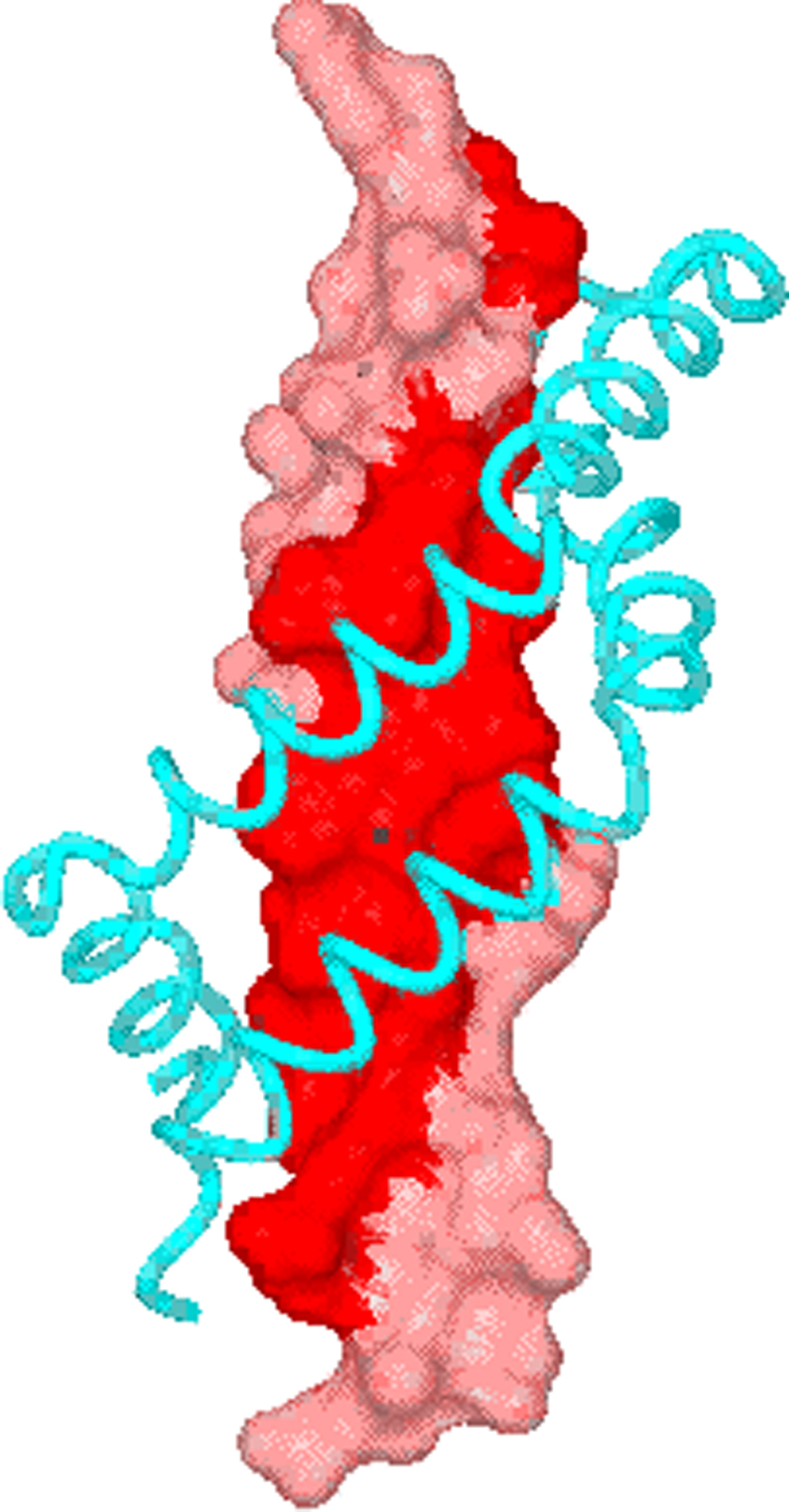

Supplement: Movie S3. Sac3CID Interface with Sus1 — Surface view of the Sac3 helix (residues 753–790; red) showing how the residues that form the interaction interface with Sus1 (Sus1B, cyan) form a helical stripe (dark red) that winds around the Sac3 α helix. [file mmc4.jpg]
